# 

**Published:** 1856-10

**Authors:** 


### INDEX.—VOL. VI.

A.

PAGE.

Alveolar Abcess, Treatment of, 141
Amalgam for filling Teeth, on the use of, . . . . . 143
Anodyne Cement, . . . 147
Adipocire, its formation, . 147
Ancients, knowledge of the teeth among, . . . . . 157
Artificial Teeth mounted on a base of Gutta Percha, . 162
Accidental Poisoning, . 164
Address to Graduating Class of Balt. Col. of Dent. Sur. By Dr. Brown, . . . . 201
Aluminium, New mode of preparing. By Prof. Rose, . 239
Ayers, P. B. Micro-chemical Researches on the digestion of starch and amylaceous foods, 276
Anæsthesia, Local, . . . 294
Anatomy, Minute, of the Liver, 309
Appointment, . . . . 313
American Medical Assoc'n, . 319
Aluminium, or Aluminum, . 320
Austen, P. H. Metallic Dies, 363
Ambler and Avery's Contributions to the World's Fair, 378
Anæsthesia, Local, by Congelation, . . . . . 465
Animal Fluids, Decomposition of Starch, by. . . . 466
Am. Jour. Science and Art, . 475
Am. Dent Convention, . 476
Artificial Teeth, India Rubber base for, . . . . 477
Adjustable Punch, . . . . 479
American Dental Convention, Second Annual Meeting of, 497
Arthur's Improved Method of using Gold Foil, . . 583
Adjusting Lower Plates, . . 599
Artificial Teeth, Preparation of the Mouth for, . . . 619
American Dent. Con. . . 633
Anæsthesia Dent., Quinton, . 632
Aluminium, . . . . 636

B.

PAGE.

Badger. F. H., Filling Teeth, 20
Blow Pipe, Improved, . 156
Beal, Dr. . . . . . 160
Badger's method of filling post. approx. cavities, . . 163
Brown, Dr. Solyman, Valedictory, . . . . . 201
Buck, Dr. Gordon, Deep wound of the Parotid Region, . 271
Bilious Remittent Fevers, Essay on, . . . . . 311
Balt. Coll. of Dental Surg. . 314
" " " " Operations in Infirmary of, . 317
Balt. Dent. Depot, . . . 321
Bucket, Valuable, . . . 324
Brachio-cephalic Artery, Hist. of the Ligature applied to. By Paul F. Eve, . . . 475
Burr, American Dent. Convention, Reported by, . 497
Brief extracts from Proceedings Penn. Assoc'n, . . . 593
British Journal of Dent. Science, 634

C

Coghlan John, Pivoting Teeth, 24
Chloroform, remarks on the employment of, . . . . 27
Continuous Gum Work, . 44
Caries of the Teeth, By Taft, 87
Caries Dental, By Taylor. 94
Chlorine. By G. Watt, . . 102
Chloroform, Means of Counteracting Effects of, . . 117
Chloroform, Death from Inhalation of, . . . . 119
Crown, New Formation of . 140
Chorea, Cases of, Cured by the Extraction of Teeth, . 146
Cement, Anodyne, . . . 147
Chloroform, Fatal Effects of, . 154
Cup, Franklin's Improved, for Impressions, . . . 156

PAGE.
Crystalline or Sponge Gold, 158
Cheiloplasty, . . . . . 159
Calculus, Salivary, . . . 164
Chemistry of Metals, Copper. By Wright, . . . . 165
Copper, . . . . . . 165
Chase, H. N., Manner of Swaging Plates, . . . . . 197
Caries, Treatment of Teeth endangered by, . . . . 211
Chloroform and its Management, 229
Chapelle, Dr., Treatment of Typhoid Fever with Tar Water, . . . . . 289
Continuous Gum Work, . 296
Carson John, Effects of Lead on the Heart, . . . . . 311
College, Baltimore Dental, 314
" Philadelphia Dental, 317
Cicatrices, Medico-Legal, Importance of . . . . . 322
Calcification, Intrinsic, of Permanent Tooth Pulp, as associated with Caries. By Salter, 337
Contributions to World's Fair, Ambler's and Avery's, . . 378
Congelation, . . . . 465
California Medical Society, 477
Convention, American Dental, 497
Caries, Dental Treatment of, by Taylor, . . . . 606
Cylindrically Prepared Foil, 625
Congelation, by Quinton, 632

D.

Dental Pulp, Treatment of Diseases of. By C. A. Harris, 3
Dental Caries, Treatment of, by J. Taylor, . . . . 94
Death from Chloroform, . 119
Duvall, W. W., Rupture of Uterus, . . . . . 120
Dental Operations, Management of Light in, . . . 142
Dental Hygiene, . . . . 143
Development, Anomalous and Union of Three Teeth, . 158
Dental College at Syracuse destroyed by Fire, . . . 163
Dental Deformities. By Levison, 184
Dental Pathology, Specimens of, 226
Dental Hygiene, . . . 231
Digestion of Starch and Amylaceous foods, by Ayers, . 276

PAGE.
Dentition in Children, connected with nursing and weening, by M. Trousseau, . . . 284
Dynamometre, . . . . 291
Dentists' Soldering Lamp, 322
Dentistry, triumph in, . 324
Disease of the Mucous Membrane of the Mouth, etc., . 325
Dies, Metallic, by Austen, 363
Dental Tissues, on certain conditions of, . . . . . 418
Dentistry in Germany, . 463
Diseases of Maxillary Sinus, 402, 481
Dental Society at Chicago, Report of the Meeting of . 563
Dentine Sensibility of, Essay on Treatment of, byJ. D. White, 578
Dental Surgeons, Penn. Association of, . . . . . 593
Dental Caries, Treatment of, 606
Dental Anæsthesia, . . . 632
Dental Convention, . . 633
Dental Western Society, 634
Dental Obturator, . . . 634
Dental Science, British Jour. of, 634
Dental Register of the West, 635
Decomposition of Iodide of Starch by Animal Fluids, . . 466

E.

Exhibition, The Paris, . . 39
Electrotype Manipulations, 121
Epidemics, Ozone in, . . 149
Essay on Intermittent and Bilious Remittent Fevers, . 311
Eve, Paul F., History of Ligature of Brachio-cephalic Artery, 475
Essay on Sensibility of Dentine, &c. . . . . 578

F.

Filling Teeth, by Badger, . 20
Filling Teeth, Amalgam for 143
Fatal effects of Chloroform, 154
Franklin's Improved Cup, 156
Food, Milk an article of, . 164
Fracture of the Hyoid and Inferior Maxillary Bones, by Sawyer, . . . . 250
Foil, Gold, Arthur, . . 583
Field, A. G. Fissure of Hard Palate, . . . . . 602
Foil, Cylindrically prepared, 625

PAGE.

G.

Gum Work, Continuous, . 44
Gutta Percha, Artificial Teeth, Mounted in, . . . . 35
Gold, Crystallized, . . 111
Gutta Percha Pencils, . . 155
Gold, Crystalline or Sponge, 158
Gutta Percha, Artificial Teeth Mounted in, . . . . 162
Glucogenic Function of the Liver, . . . . . 213
Gum Work, Continuous, . 296
Gaillard, M., Intermittent and Bilious Remittent Fevers, 311
Gold Filling removed from the Neck, . . . . . 323
Giraldes, Dr., Diseases of Maxillary Sinus, . . 402, 481
Gums, Hypertrophy of, . . 626

H.

Harris, Chapin A., Diseases of Dental Pulp, . . . . 3
Hays, George, Sketches of the Knowledge of Teeth, . 189
Holmes, Alex., Specimens of Dental Pathology, . . 226
Hyoid and Inferior Maxil. Bones, Fracture of, . . . 250
Howard, Dr., Ascent of Mont Blanc, . . . . . 311
Heart, Effects of Lead on, 311
Handy, W. R., Importance of a more thorough knowledge of Dental Organs on the part of Physicians, &c. . . . . 387
Hypertrophy of the Gums, 626
Hogg, Jabez, Microscope, History, Application, &c. . 630

I.

Iris, Influence of the Spinal Cord on the Movements of the, 307
Iodide of Starch, Decomposition, 466
India Rubber Base, for Artificial Teeth, . . . . . 477
Introductory Lecture, . 480

J.

Johnston, Dr. Chris., Diseases of Maxillary Sinus, Translated by, . . 402, 481

PAGE.

K.

Knowledge of Teeth among the Ancients, . . . . . 157

L.

Light in Dental Operations, 142
Light, Management of in Dental Operations, . . 145
Levison, J. L., Dental Deformities, . . . . . 184
Liver, Minute Anatomy of, 309
Lead, Effects of on the Heart, 311
Liver, Glucogenic Function of the, . . . . . 213
Louisville Review, . . 475
Ligature applied to Brachiocephalic Artery, . . 475
Lining Teeth, . . . 492

M.

Milk, an article of Food, . 164
Mercurial Stomatitis, by Reynolds, 220
Maxillary Inferior, Fracture of, 250
Micro-Chemical Researches, 276
Memoir on the Loss of the Teeth, by Dr. Rossi, 289, 453
Mucous Membrane of the Mouth, Disease of, . . . 325
Maxillary Sinus, Diseases of, 402, 481
Maxilla, Necrosis of Inferior, 437
Mouth, Reparation of, for Artificial Teeth, . . 619
Microscope, History, Construction and Application, . 630

N.

Nott and Gliddon, Types of Mankind, . . . . . 45
Nerves, Spinal, . . . . 308
New York Teeth Manufacturing Co. . . . . . 321
Necrosis of Inferior Maxilla, 437
New York Journal of Medicine, 636

O.

Ozone in Epidemics, . 149
Ozone and Cholera, . . 153
Osseous Union of three Teeth, 158
Origin of Medical Science, 189
Ozone, . . . . . 311

PAGE.
Operations in Infirmary of Baltimore Col. of Den. Sur. 317
Osseous Union of Teeth, . 463
Osseous Union of Teeth, 480
Obturator, Dental, . . 634

P.

Pivoting Teeth, by Coghlan, 24
Paris Exhibition, . . . . 39
Platinum or Continuous Gum Work, . . . . . 44
Pancreatic Juice, . . 306
Philadelphia Dental College, 317
Physiology of Spinal Cord, · 470
Pulp, Treatment of Exposed, 606

Q

Quinton, Richard, Painless Tooth Extractor, . . 632

R.

Reynolds, R. Bruce, Report of a Case of Mercurial Stomatitis, . . . . . 220
Rose, Prof., Mode of Preparing Aluminium, . . . . 229
Rossi, Dr., Memoir on the Loss of Teeth, . . 298, 453

S.

Slayton, N. B., Artificial Teeth mounted on Gutta Percha, 35
Springing of Plates, . . 145
Salivary Calculus, . . 164
Sawyer, Albert, Fracture of the Hyoid and Inferior Maxillary Bones, . . . . . 250
Spleen, Functions of, . . 302
Saliva, . . . . . 307
Spinal Cord and Spinal Nerves, Structure of. . . . . 308
Salter, S. James, on Intrinsic Calcification of the Permanent Tooth Pulp, . . . . 337
Steel, Working of, . . . . 464
Spinal Cord, Physiology of, 470
Smith, H. R. Preparation of the Mouth for Artificial Teeth, 619

PAGE.
Saliva, . . . . . 636

T.

Taft, J., Caries of the Teeth, 87
Taylor James, Treatment of Dental Caries, &c. . 94, 606
Townsend, E., Lecture on the duties, &c. of Dental Practitioners, . . . . . 56
Townsend, E., Lecture on Generalities of Professional Conduct, . . . . . . 72
Trousseau, M., Connection of Dentition in Children with Nursing and Weaning, . 284
Tape Worm, . . . . 307
Teeth, Disease of, caused by disease of Mucous Membrane of the Mouth, . . . . 325
Tyros, Dental Qualifications for, 356
Tomes, John, on Conditions of Dental Tissues, . . 418
Teeth, memoir on the loss of 298, 453
Teeth, Artificial base for, . 477
Teeth, Supernumerary, . 480
Teeth, Lining of, . . 592
Teeth, Artificial, preparation of the Mouth for, . . . 619
Teeth Manufacturing Co. . 321

U.

Uterus, Case of Rupture of, 120
Ulcerations, Phagedenic, . 146

V.

Valedictory Address. By Solyman Brown, . . . 201

W.

Watt, G., Chlorine, . . . 102
Wright, R. M., Chemistry of the Metals, Copper, . 165
"Wax," Problem, . . 237
Wood, James R., Necrosis of Inf. Maxilla, . . . . 437
Western Dent. Society, 477, 563, 634
White, J. D., Sensibility of Dentine, . . . 578

